# Bacteriophage therapy: a possible alternative therapy against antibiotic-resistant strains of *Klebsiella pneumoniae*

**DOI:** 10.3389/fmicb.2025.1443430

**Published:** 2025-03-31

**Authors:** Sadia Abbas, Rabia Kanwar, Kaleem Ullah, Rimsha Kanwal, Mamoon Tajamal, Muhammad Aamir Aslam, Abid Ahmad, Abdul Qadeer, Hsun-Yu Huang, Chien-Chin Chen

**Affiliations:** ^1^Institute of Microbiology, University of Agriculture Faisalabad, Faisalabad, Pakistan; ^2^Directorate General (Research) Livestock & Dairy Development Department Peshawar, Peshawar, Khyber Pakhtunkhwa, Pakistan; ^3^Department of Animal Nutrition, The University of Agriculture Peshawar, Peshawar, Khyber Pakhtunkhwa, Pakistan; ^4^Department of Cell Biology, School of Life Sciences, Central South University, Changsha, China; ^5^Division of Endodontics, Department of Stomatology, Ditmanson Medical Foundation Chia-Yi Christian Hospital, Chiayi, Taiwan; ^6^Department of Pathology, Ditmanson Medical Foundation Chia-Yi Christian Hospital, Chiayi, Taiwan; ^7^Department of Cosmetic Science, Chia Nan University of Pharmacy and Science, Tainan, Taiwan; ^8^Doctoral Program in Translational Medicine, Rong Hsing Research Center for Translational Medicine, National Chung Hsing University, Taichung, Taiwan; ^9^Department of Biotechnology and Bioindustry Sciences, College of Bioscience and Biotechnology, National Cheng Kung University, Tainan, Taiwan; ^10^Biotechnology Center, National Chung Hsing University, Taichung, Taiwan

**Keywords:** *K. pneumoniae*, antibiotic resistance, bacteriophages, phage therapy, endolysins

## Abstract

*Klebsiella pneumoniae* is a notorious, Gram-negative pathogen and is a leading cause of healthcare settings and community-acquired infections. This is the commensal of human microbiota and can invade and cause infections in different body parts. The global emergence of antibiotic resistance in *K. pneumoniae* has become a major challenge in the whole medical community. Alternative paths to treat the infections caused by these MDR pathogens are needed as these bacteria become resistant to last-resort antibiotics like colistin. The lytic bacteriophages (phages) are the bacteria's natural predators and can rapidly eliminate the bacterial cells. Phages are abundant in nature and have recently been found to be effective tools in modern biotechnology. They can be used to control the bacterial infectious diseases. They can be manipulated easily and potentially used in therapeutics, biotechnology, and research. Several studies, both *in vitro* and *in vivo*, have demonstrated the possible applications of the lytic phages in treating *K. pneumoniae* superbug strains. Phage endolysins have drawn the scientific world's attention because of their involvement in phage adsorption and bacterial capsules digestion. These phage-encoded enzymes digest the polysaccharide components of bacterial cell walls by recognizing and binding them. Phage lysins, being strong biological agents, are capable of effectively and swiftly eliminating bacteria. This review summarizes the information on phages of *K. pneumoniae* and phage-based therapies to target their bacterial hosts.

## 1 Introduction

*Klebsiella pneumoniae* is a facultative, anaerobic, opportunistic pathogen of the Klebsiella genus of the Enterobacteriaceae family that was first isolated from the lungs of pneumonia patients by Carl Friedlander in 1882. They are rod-shaped, non-motile, and encapsulated bacteria (Mohammadi et al., 2023a). *K. pneumoniae* is ubiquitous in water, soil, sewage, plants, and animals and frequently colonizes mucosal surfaces, invades, and causes infections in different body sites. The ability of this pathogen to evade the immune system, its increasing antibiotic resistance, and the spread of hypervirulent strains has become a challenge in healthcare settings (Choby et al., 2020; Ballén et al., 2021). Immunocompromised patients, neonates, and the elderly are more vulnerable to infections. Bacterial infections are more common in healthcare settings and spread via direct contact with an infected person (Santajit and Indrawattana, 2016). It is mainly responsible for urinary and respiratory tract infections and wound and soft tissue infections and is extensively present in medically used devices such as ventilators, catheters, contaminated surfaces, and other instrumentations (Yan et al., 2016; Maebed et al., 2021). It is the second most common pathogen after *E. coli* that causes nosocomial and community-acquired infections (Moya and Maicas, 2020). This pathogen belongs to the ESKAPE group, which includes the *Enterococcus faecium, Staphylococcus aureus, Klebsiella pneumoniae, Acinetobacter baumannii, Pseudomonas aeruginosa* and *Enterobacter* species that are mainly responsible for the majority of nosocomial infections and can escape from the biocidal activity of the antibiotics (Hoenes et al., 2021; Sethuvel et al., 2023). *K. pneumoniae* follows the model of “the best defense for a pathogen is a good defense” instead of “the good defense for a pathogen is a good offense.” That ability makes the bacteria escape and sustain their life in the host. The hyper-virulent strains of *K. pneumoniae* are more virulent compared to classical *K. pneumoniae* strains and cause community-acquired infections even in healthy individuals, and are more difficult to control and treat due to high occurrence at the multiplication sites and subsequent spread (Holt et al., 2015). Hypermucoviscous *K. pneumoniae can* metastatically spread from one organ to another organ, which is rare for enteric Gram-negative bacilli in the presence of host immunological defense. Capsule polysaccharide (CPS) protects the bacteria and has a role in the resistance to antimicrobial peptides, phagocytosis suppression of early inflammatory response, and inhibition of DC maturation (Paczosa and Mecsas, 2016). The hypervirulent *K. pneumoniae is* responsible for pyogenic liver abscesses, meningitis, endophthalmitis, and septic arthritis (Choby et al., 2020).

In recent years, antimicrobial resistance has become a major challenge and causes a variety of nosocomial infections that can be problematic (Amraei et al., 2022). The majority of the antibiotic-resistant mechanisms have been acquired through horizontal gene transfer, imparting a high level of resistance to β-lactam and quinolone classes of antibiotics (Mohammadi et al., 2023a). Due to the growing worldwide problem of antibiotic resistance, the World Health Organization recognizes *K. pneumoniae* as a species of major importance and encourages the development of new antibiotics and other treatment options (Tacconelli, 2017). One of the most promising alternatives to antibiotics is the use of bacteriophages and phage-based products (Czaplewski et al., 2016). Bacteriophage therapy is described as administering whole lytic phage or purified phage particles to a patient to lyse the bacterial pathogen causing the infection (Kutter et al., 2010). it is a promising strategy to combat the infections caused by antibiotic-resistant bacteria. Antibiotic resistance and antibiotic-sensitive bacteria are both effectively treated using phage therapy. Their function is bactericidal rather than bacteriostatic, killing their target pathogens and suppressing bacterial development toward resistance (Chan et al., 2018).

Bacteriophages are the bacterial predators and obligate intracellular parasites that lyse the bacteria and have the advantage of being highly specific to their bacterial hosts, which reduces the risk of disrupting the natural microbiome and causing collateral damage to healthy cells (Pirnay et al., 2015). Bacteriophages, due to their high diversity, are found in a variety of environments, such as the human gut, soil, sewage water, and aquatic systems (Zhu et al., 2022). They are 10 times the number of bacteria present in the environment and have multiple applications due to their lifestyle and unique properties. Bacteriophage degrading enzymes, as antibacterial agents, are becoming more and more desirable, particularly against antibiotic-resistant pathogens, and phage-associated lysins and endolysins have become the prominent new antimicrobials. As recombinant proteins, the usage of purified endolysins to lyse the bacteria was documented in 1959. Endolysins are highly evolved enzymes that efficiently lyse the bacterial host cell wall by hydrolyzing the four primary bonds in peptidoglycan components, causing the cell wall to break (Abdelrahman et al., 2021). They work in association with holin proteins and provide the lysins access to the peptidoglycan layer by perforating the inner cell membrane. Because the peptidoglycan layer gives the cell its structural integrity and rigidity, its degradation causes the cell wall to become unstable and eventually rupture because of variations in the osmotic pressures inside the cell and in the environment. Endolysins administered exogenously can have a direct impact on specific Gram-positive bacteria because they lack the outer membrane. In the case of Gram-negative bacteria, endolysin-induced lysis has just evolved and requires spanin complexes for the outer membrane disruption, which catalyzes the outer and inner membrane fusion. Several studies have been conducted on the efficacy of phage lysins in treating systemic and mucosal infections to determine the validity of their bactericidal potential (Belay et al., 2018).

## 2 Pathogenesis and antibiotic resistance

**Figure 1:**
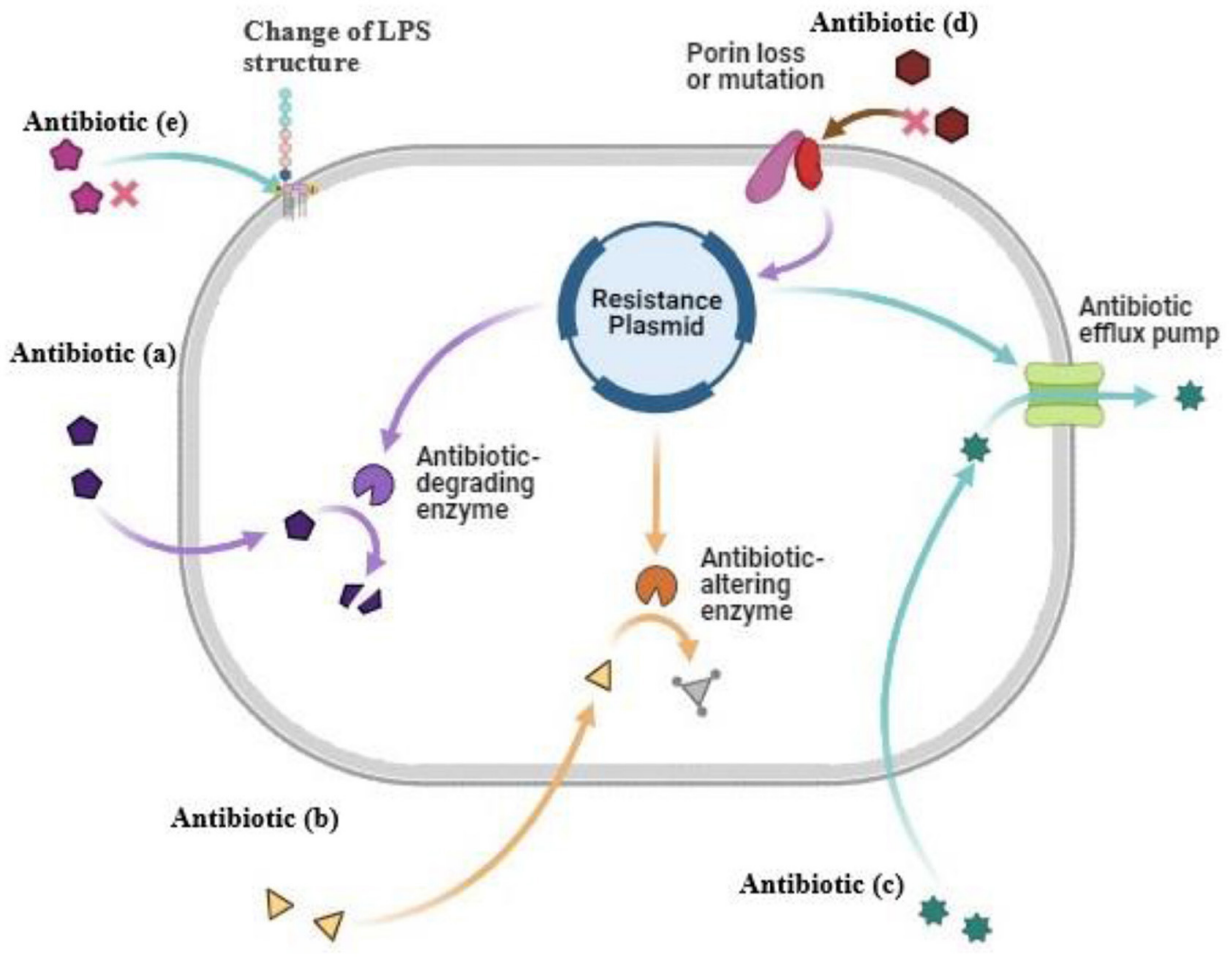
Antibiotic resistance mechanism in K. pneumoniae. **(a)** Antibiotic degrading enzymes degrade the antibiotic **(b)** Antibiotic altering enzymes alter modified the antibiotic **(c)** Antibiotic efflux pump cause pumping out of antibiotic before it reaches the target **(d)** Modification in cell wall porins **(e)** Change of LPS structure of cell wall.

A large spectrum of bacterial components provide the virulence attributes of encapsulated *K. pneumoniae* that lead to infections and antibiotic resistance (Figure 1). Lipopolysaccharide (LPS), capsule polysaccharide (CPS), fimbriae, and siderophores (Ali et al., 2022), including enterobactin and aerobactin, are the critical virulence factors of *K. pneumoniae*. A few virulence factors, like capsule, fimbriae, and biofilm formation, are the origin of classical pathogenesis and are present in nearly all isolates. LPS is composed of three important components: Lipid A, core, and O antigen. The host immune system initiates the cascade of reactions by identifying the LPS, but the pathogen can modify the LPS in parts that are not recognized by the host immune cells, allowing it to successfully uphold the infection (Llobet et al., 2015). The CPS is essential in the escape of *K. pneumoniae* from the host. The CPS is the top protective layer engaged in the initial interaction between the host and the bacteria. The CPS locus contains several genes that are critical for the synthesis of CPS. The biosynthesis of CPS is mediated by the RmpA gene, a characteristic of hvKP, and is a plasmid-located virulence factor. The binding and adherence of the infectious agent to the host mucosal surface enable it to survive and establish the infection inside the host, and the bacterial component, which helps it to adhere to the host cell surface, is the fimbriae or pilli, and it is an important virulence factor for the bacteria (Navon-Venezia et al., 2017). During infection, like other human pathogens, *K. pneumoniae* requires iron molecules from its host cell as it is very critical for propagation and growth. For this purpose, siderophores play an important role and have a high affinity for iron binding. *K. pneumoniae* is a significant contributor to antibiotic resistance in addition to being widely prevalent, as it shows the non-susceptible rates against the four major classes of antibiotics, which include the carbapenems, quinolones, aminoglycosides, and third generation cephalosporins. It emphasizes the significant contribution of this pathogen to the worldwide burden of antibiotic resistance. A major class of antibacterial agents used in human medicine is the β-lactam antibiotics that have been clinically used since the 1940s, and it includes penicillin, carbapenems, cephalosporins, monobactam, and β-lactamase inhibitors, these are most widely used antibiotics with a β-lactam ring in their molecular structure. The β-lactamase production is the first defense strategy adopted by *K. pneumoniae* to resist the antibiotics, which is followed by alteration in permeability. The β-lactamase genes, SHV-1 and TEM-2, were identified as a result of penicillin resistance in 1960 in *K. pneumoniae* (Wang et al., 2020). Due to the presence of SHV-1 β-lactamase encoded on the chromosome, *K. pneumoniae* is resistant to carbenicillin and ampicillin. Shortly after, an ESBL gene SHV-2 and another plasmid-mediated ESBL gene TEM-3 from this infectious agent were reported. Carbapenemase production, activation of efflux pumps and ESBLs, and AmpC cephalosporinases overexpression with less expression of OMPs are the main carbapenem-resistant mechanisms. The decreased susceptibility to carbapenemase is also associated with disruption of OmpK35, and OmpK36. In *K. pneumoniae*, the IMP-1 metallo-enzyme was the first detected carbapenemase that was reported in Japan in 1991. Undoubtedly, blaKPC became the highly impacting carbapenemase, with blaKPC-2 and blaKPC-3 being the most frequent genes (Lee et al., 2016), and incredibly common in strains linked to hospital outbreaks in many different countries. The DNA replication is inhibited by the Quinolone antibiotics that target the bacterial topoisomerases. The most frequent use of the quinolones caused its resistance (Naeem et al., 2016). The chromosomal mutations in DNA gyrase, quinolone binding targets, and topoisomerase IV having parC-parE subunits are the major resistance mechanisms, but the more common mutations in gyrA and parC were recognized earlier in *K. pneumoniae* than mutations in gyrB and pare (Guillard et al., 2016). Aminoglycosides, protein synthesis inhibitors, were replaced by fluoroquinolones, carbapenems, and third-generation cephalosporins due to high antibiotic resistance (Krause et al., 2016) gained through mechanisms involving drug modification enzymes with acetylation, adenylation, or phosphorylation activities (Peirano et al., 2014). The other resistance mechanisms in *K. pneumoniae* against aminoglycosides include loss of KpnO, a putative porin, and cell permeability modifications because of changes in AcrAB-TolC and KpnEF efflux pump systems (Padilla et al., 2010). Table 1 represents the data of antibiotic resistance genes in *K. pneumoniae*.

**Table 1:** Antibiotic resistance genes in *Klebsiella pneumoniae*.

**Antibiotics**	**Resistant genes**	**Functions**	**References**
β**-lactams**			
Class A	KPC	Serine β-Lactamase, Carbapenemase	(Boyd et al., 2020; Zhuang et al., 2021)
Class B	NDM	Carbapenemase	(Sawa et al., 2020)
Class C	VIM	Carbapenemase	(Sawa et al., 2020; Boyd et al., 2020)
Class D	OXA-48	Cleaves Oxacillin besides penicillin, Carbapenemase	(De Angelis et al., 2020)
	pmrE, pbgP	Amino arabinose combination	(Aslan and Akova, 2022)
	CTX-M	Iatrogenic outbreaks led to ESBL in Klebsiella pneumonia	(Alfei and Schito, 2022)
	blaGES, blaVEB and blaOXA	Acquisition of horizontal gene transfer	(Li et al., 2018; Alfei and Schito, 2022)
	pmrC	Phosphoethanolamine combination	(Aslan and Akova, 2022)
	ramA, IpxM	Maturation and neutralization of lipid A	(Lv et al., 2021)
Quinolones	Qnr	Encoding the proteins family that shield topoisomerase IV and DNA gyrase	(Bush et al., 2020)
	blaTEM-3	plasmid mediated ESBL variant	(Kalali et al., 2022)
	kdeA	Permeability of cell	(Lv et al., 2021)
	blaSHV-1	Resistant to penicillin	(Hussain et al., 2021)
	blaSHV-2	ESBL gene	(Hussain et al., 2021)
Aminoglycosides	OmpK36	Permeability of cell	(Lomovskaya et al., 2022)
	gyrA-gyrB subunit	Ofloxacin and nalidixic acid resistance	(Nam et al., 2013; Tang and Zhao, 2023)
	parC-parE subunit	Ofloxacin and nalidixic acid resistance	(Bush et al., 2020; Tang and Zhao, 2023)
	acrAB	Permeability of cell	(Lv et al., 2021)
Polymyxin	pmrA, phoPQ	Gene regulators modified by LPS	(Dalmolin et al., 2018; Shahzad et al., 2023)
	mgrB, pmrD	Gene regulators modified by LPS	(Shahzad et al., 2023)
	RamA, AcrR	Efflux pump regulators	(Osei Sekyere et al., 2016)
	Mrc-1	Via plasmid, promotes the addition of Phosphoethanolamine to lipid A	(Zhu et al., 2022)
Tetracycline	tetA, tetC, tetG, tetK	Responsible for energy dependent efflux pump	(Zhuang et al., 2021; Xu et al., 2022) (Jian et al., 2021)
	tetM, tetO, tetQ, tetw	Ribosomal protection proteins	(Jian et al., 2021; Xu et al., 2022)
	tetX	Involve in Enzymatic inactivation	(Xu et al., 2022)
Sulfonamide	sul1, sul2, sul3	Correlated with mobile genetic elements	(Kunhikannan et al., 2021)

## 3 Phages of *Klebsiella pneumoniae*

The therapeutic use of phages against bacterial infections has gained momentum due to the emergence of antibiotic resistance in bacteria such as multi-drug resistant *K. pneumoniae*. Felix d'Herelle was the first to propose the therapeutic use of bacteriophages in 1917 to treat the infections of clinically important pathogens in humans and animals. Several factors are involved in the selection of bacteriophages for bacteriophage therapy. Firstly, phages should continue to be effective in eliminating the *K. pneumoniae* (Fang and Zong, 2022; Habibinava et al., 2022). The host range, strictly lytic nature, stable and able to replicate in the host, synergistic therapeutic effects coupled with antibiotics and other phages in the form of a phage cocktail to inhibit the development of antibiotic resistance (Al-Ishaq et al., 2020). When evaluating the phages, *in vitro* analysis of phage lysis is done by incubating phage with cultures of *K. pneumoniae* (Hesse et al., 2021). Several mouse models have been established to investigate the phages of *K. pneumoniae*, such as sepsis (Vinodkumar et al., 2005), lobar pneumonia (Chhibber et al., 2008), liver abscesses (Hung et al., 2011), and burn wound infections (Malik and Chhibber, 2009). The therapeutic efficacy of tailed bacteriophage BPA43 was assessed in a BALB/c pneumonic mouse model, which was infected with virulent *K. pneumoniae*. There was a great reduction in the bacterial count in the lungs of mice after 2 h of post-challenge with *K. pneumoniae* (Anand et al., 2020). The bacteriophage Kpn5 against *K. pneumoniae* B5055 was effective in treating burn wound infections in murine and mice models when compared with silver nitrate and gentamicin (Kumari et al., 2009). Phage KPO1K2, encapsulated within a liposome, was found to be efficacious in treating lobar pneumonia in BALB/c mice caused by intranasal inoculation of *K. pneumoniae* B5055. Phage therapy was also postponed by around 3 days. This highlights the need to research multiple transport techniques for phage treatment, not just from a logistical aspect but also in determining the most efficient strategy based on the type of contamination and the depth of incubation in compliance with the treatment (Chadha et al., 2017). The use of a phage cocktail (made up of Kpn1, Kpn2, Kpn3, Kpn4, and Kpn5) caused a decrease in bacterial load. A mouse infected with *K. pneumoniae* ST258 was rescued by combined phage therapy that showed the least phage resistance frequency and more survival rate (Hesse et al., 2021), another phage cocktail (Katrice−16) made up of eight lytic phages was identified for its potential use against 56 strains of *K. pneumoniae* ST16. The Katrice-16 was highly active in *in-vitro* as well as in human fluid (Martins et al., 2022) against *K. pneumoniae*, which is mostly responsible for burn wound infections. Many phages have been used against that like phage KØ1, phage P?Bw-Kp1, phage P?Bw-Kp2, phage P?Bw-Kp3 (Torabi et al., 2021), a lytic phage vB-KpneM-Isf48 (Komijani et al., 2017), and phage ZCKP8 (Fayez et al., 2021). A phage cocktail of three lytic phages named GH-K1, GH-K2, and GH-K3 was specific to *K. pneumoniae* strain K7 (Wu et al., 2019). Multiple studies have found that combining antibiotics and phages has a greater antibacterial impact than either drug alone (Viertel et al., 2014). The synergistic effects of phage with antibiotics established the more favorable effects, such as the combination of ceftriaxone with phage vB-1086 showing great activity to inhibit biofilm formation (Xu et al., 2022). Similarly, phage-ciprofloxacin and phage-meropenem synergism are also effective in impeding biofilm formation compared to individual treatments (Shi et al., 2020). Furthermore, choosing effective phages for the bacteria to be targeted can boost phage performance, phage cocktails are used to improve the impact of phages on *Klebsiella* populations and are considered crucial for the efficacy of phage therapy (Chan et al., 2013). Table 2 shows the data of some recently isolated phages against *K. pneumoniae*.

**Table 2:** Some recently isolated bacteriophages against *K. pneumonia*e.

**Bacteriophage**	**Experimental design**	**Results**	**Reference**
KP1LMA	Sterile urine was inculated with phage (10^6^ PFU/ml) and bacteria 10^5^ CFU/mL to generate a UTI model.	3.8 log CFU/mL of bacterial inactivation was observed	(Duarte et al., 2024)
Kp7450ΔfabF	Phage antibacterial activity was measured with mutant strain of bacteria in Zebrafish model	LPS significantly impacted the virulence of bacteria. Phage Kp7450ΔfabF showed lytic activity against mutant strain.	(Li et al., 2023)
Phage M1	Phage with antibiotics meropenem, colistin, and ceftazidime was given to a 30 years old patient with wound infection.	Phage in synergism with antibiotic has significantly reduce the bacterial load and improved patient's condition	(Eskenazi et al., 2022)
EKq1	Isolated against blood borne MDR *K. pneumoniae*.	The phage could have potent antibacterial activity against MDR *K. pneumoniae*.	(Bird et al., 2024)
vB_KshKPC-M	*In-vitro* evaluation of phage against carbepenem resistant *K. pneumoniae* was determined.	Phage vB_KshKPC-M have broad host range and could be a potent in treatment of carbapenem resistance *K. pneumoniae*.	(Mohammadi et al., 2023b)
KPW-1, KPW-2, and KPW-3	*In-vitro* evaluation of isolated phages and their potent anti-biofilm activity was measured	Cocktail of these isolated phages significantly inhibit the bacterial growth and biofilm.	(Wei et al., 2024)
PG14	*In-vitro* anti-biofilm activity of isolated phages.	80% reduction in bacterial biofilm	(Mulani et al., 2022)
vB_KpnS_FZ10, vB_KpnP_FZ12, and vB_KpnM_FZ14	Cocktail of three phages. Each phage with concentration of 10^7^ PFU/ml was given.	Efficiently reduced the number of bacterial cells and biofilm formation.	(Zurabov et al., 2023)
vB_Kpn_ZCKp20p	Anti-biofilm activity And toxic effect human fibroblast was measured	Inhibit the biofilm formation at lowest MOI 0.01. No toxic effect was observed on human fibroblast cell line.	(Zaki et al., 2023)

## 4 Characterization of phage

Comparing genomic sequences of *Caudovirales*, the lytic *K. pneumoniae* phages reveal some similarities and differences. For instance, there is a possibility that recently identified phages of *K. pneumoniae* polysaccharide depolymerase are involved in breaking down the outer layer that surrounds the bacterium (Kesik-Szeloch et al., 2013; Fokine and Rossmann, 2014; Jamal et al., 2015). The degradation by phage depolymerizes that enable *K. pneumoniae* to eradicate its biofilm (Taha et al., 2018) as well as make it prone to killing with antibiotics, phages, and immune clearance (Kesik-Szeloch et al., 2013). Furthermore, in the laboratory, depolymerase activity becomes apparent by lytic haloes produced around clear zones of lysis on pyoverdine plates after inoculating *K. pneumoniae* along with phage particles. This is now the basis for many novel phage laboratory methods, which include defining the specificity of a given phage against its host range (Hughes et al., 1998).

However, for initial identification, *Ackermannviridae, Myoviridae, Podoviridae*, and *Siphoviridae* differences can be helpful. Restriction analysis that involves the cleavage of phage DNA by bacterial restriction enzymes can also assist with the determination of phage genome size in addition to having some of the useful characteristics of the known phages before in-depth characterization, and it is possible to reveal the presence of forms of the phage tails with the help of transmission electron microscope. The phylogenetic analysis revealed that various phages of *Klebsiella* belong to the recognized genera within the *Siphoviridae, Myoviridae, Podoviridae*, and *Ackermannviridae* families, whereas others are rooted in novel lineages without an official viral classification (Kesik-Szeloch et al., 2013).

## 5 Host range and specificity

Specifically, they must be able to attach to a specific target bacterial host cell before initiating the lytic phase of its life cycle. It achieves this by first identifying and attaching itself to a receptor on the host cell's outer membrane. It is there where the phage receptor will interact with host receptor, which allows accessibility to infected bacteria and guides them for viral DNA transfer into their stealer cell. Adsorption can occur on any outer structure in different phages to host but in case of gram-negative bacteria such as *K. pneumoniae* adsorption may be either by pili, capsule, LPS or bunch of other structures outside the bacterial cell wall (Bertozzi Silva et al., 2016). Flu phage1/9 binds at any of these three sites on the host's exterior to infect its *E. coli* hosts; so, it sets boundaries for what we can call as a “host” (D'Andrea et al., 2017). Further illustrated the fine specificity of their modern lytic phage ϕBO1E which exclusively lysed KPC-producing (i.e., clade II) but no other strains with only clade I species showing that there was inherent sensitivity and concurred and did not kill strains within clade I because it recognizes some capsular polysaccharides that are unique among strains from only one major subgroup. However, their experiment showed that KPO1K2 targeting the *K. pneumoniae* B5055 is effective against multiple isolates of *E. coli* in addition to several influenzas of both species also indicating a wider scavenger range than clade selective ϕBO1E (Verma et al., 2009a).

As for their therapeutic application, it has been accepted that lytic phages with wide host specificity on the species level are more advantageous in combating bacterial infection than phages with limited host specificity on the strain level. Such targeted phages are ineffective for hypothetical or prophylactic treatment and would make use of infective agent detection before administration of phages. Moreover, even if phages to be used were to be classified as being broad host range phages, these phages would, in the general sense, be more specific than antibiotics (Loc-Carrillo and Abedon, 2011). Therefore, broadening the phage treatment's range of activity has led to the concept of phage cocktails, that can increase the number of phage particles in a single treatment (Gu et al., 2012), and even modification of the factitious host range by merging the tail structures of different bacteriophages (Yosef et al., 2017).

## 6 Potential therapeutic interests of *Klebsiella pneumonia* phages

The potential therapeutic benefits of phages that infect *K. pneumoniae* have garnered significant interest. Several factors should be considered when selecting phages for therapeutic purposes. First thing, phages must be capable of effectively eradicating *K. pneumoniae. In vitro* appraisals of burst size and phage lysis are conducted on *K. pneumoniae* cultures during the phage characterization, phages that can cause the rapid lysis and release such several particles will generate clear, sizeable plaques. Furthermore, phages having a large host range are often considered more favorable compared to ones with a small host range to target multiple strains at once (Harper, 2018). Second thing, lytic phages have the capability to clear the bacteria rapidly and effectively due to their life cycle unlike lysogenic phages, which remain dormant by incorporating the genetic information in the host's genome for an undetermined amount of time. Furthermore, the lysogenic phage can potentially transfer genes into the bacteria that enable them to produce toxins and exhibit antibiotic resistance, exacerbate the infection, and make treatment more challenging (Harper, 2018).

### 6.1 *In-vivo* testing

Prior to human trials, any therapeutic candidate must first undergo safety and efficacy testing in an appropriate animal or insect model based on *in vitro* research. The efficacy of phage treatment toward diseases such as wounds and infections of soft tissues (Kumar et al., 2010), bacteremia (Vinodkumar et al., 2005), and pneumonia (Cao et al., 2022) was researched using mouse models in the instance of *K. pneumoniae* phage, which closely imitates the range of diseases caused by the bacteria in humans. Simultaneously, the effectiveness of the lytic phages and phage-encoded molecules in eradicating infections caused by *K. pneumoniae* has been tested using *Galleria mellonella* larvae (Manohar et al., 2018). Several murine-based experiments have been conducted by Kumari and colleagues in an effort to determine the *K. pneumoniae* phage Kpn5 potential for therapeutic use. Among the five phages, isolated from sewage material (Kumari et al., 2010), Kpn5 was found to be most efficient in BALB/c mice models, unlike the other four phages when used against *K. pneumoniae* B5055 (Kumari et al., 2009) to treat burn wound infections. Kpn5 was administered intraperitoneally and resulted in an average survival rate of 9.66% compared to 0% for the negative controls. In addition, Kpn5 was found to be superior when compared to topical treatment with both antimicrobial agents (gentamicin and silver nitrate) (Kumari et al., 2011) and natural products (Aloevera gel and honey) (Kumari et al., 2010) offering reduced mortality rate and higher protection levels. However, irrespective of the promising results, the authors acknowledge the probability that *K. pneumoniae* acquiring resistance against Kpn5, as pointed out in the *in vitro* trials (Cao et al., 2015). However, they did not provide information regarding the host range of phage by using only one *K. pneumoniae* strain all over their investigations.

Another important aspect to take into account is the phage delivery method in the phage treatment. For instance, intravenous injection is used in the majority of cases in human treatment, unlike intraperitoneal injection, which is not often employed. Cao et al. (2015) conducted the experiments to treat murine-lobar pneumonia and found that intranasal administration of phage 1513 could develop an 80% survival rate in Swiss Webster mice, 2 h after nasal delivery of MDR *K. pneumoniae* 1513, compared to 0% in negative controls, in addition, noticeably decreased lung injury. The intraperitoneal inoculation of phage SS into BALB/c following intranasal administration of *K. pneumoniae* B5055, as determined (Chhibber et al., 2008) caused complete elimination of the bacteria in five days rather than 10 days in mice without treatment. Even though a 6 h delay post-inoculation made the treatment ineffective, according to the author's demonstration, Singla et al. (2015) exhibited that a liposome enclosed KPO1K2 phage was efficient for the treatment of lobar pneumonia induced by intranasal administration of *K. pneumoniae* B5055, regardless the therapy was postponed for up to 3 days, in BALB/c mice. Although the comparison cannot be made directly among studies because of the differentiations in the phage selection in the published studies, it emphasizes the significance of examining various phage therapy delivery strategies. This is not just important from an economical perspective moreover for determine the most effective mechanism based on the kind of infection and duration of incubation before therapy. Furthermore, the investigations just examined the in vivo impact of phage treatment on a single *K. pneumoniae* strain and did not provide information about the host range of phages. Consequently, more research should focus on determining if the corresponding phages' host ranges are sufficient for their potential therapeutic interests.

Even though several investigations have demonstrated that *K. pneumoniae* phages can effectively eradicate infection in Galleria and murine models, it is now necessary to consider the impact of phage infection on the microbial community (i.e., metabolome, gut microbiota) when evacuating phages (either alone or in phage cocktail) patient decontamination measure. A common bacterial population in the human gut showed a rise in phage resistance (28 to 68%) upon infection with lytic phages, as shown by Hsu et al. (2019). This observation revealed the phage infection impact on the system, as it led to quantitative changes in both susceptible and resistant isolates. Instead of eliminating the target species, phage infection altered the ecosystem to create a more hospitable gut environment. The effectiveness of phages that target *Enterococcus feacalis* was lessened in altering the microbiota, in contrast to the phages of *E. coli* and *Clostridium sporogenes*, which significantly decreased the population of *Bacteroides vulgates, Proteus mirabilis*, and *Parabacteroides distasonis* by 106g-1 stool and the populations of *Akkermansia muciniphila* and *B. fragilis* by 108g-1 stool. Phage-induced perturbation of the microbiota had an impact on the metabolome as well. In the presence of phages, the abundance of 17% of the compounds under investigation changed considerably. Hsu et al. detected that during the initial phage infection, two-fold decrease in tryptamine, the metabolite that is microbiome-associated and helps in gastrointestinal transport acceleration in mice. The finding suggests that systemic health can be affected by targeting microbiome modification by phage infection.

## 7 Practicality of phage therapy

The practical challenges related to phage therapy in developed countries like the EU and the UK represent a significant obstacle that requires attention. Currently, the safety, manufacturing, and applications of virus-based infection control techniques remain largely unregulated. The most recent attempt to address these regulatory challenges was a phase II clinical trial supported by the European Commission. This clinical trial known as PhagoBurn, was first anticipated as a multicenter, randomized, single-blind, and controlled clinical trial of phage therapy conducted between 2013 and 2017. While adhering to Good clinical trial (GCP) and good manufacturing practices (GMP) standards. Phagoburn aimed to evaluate the efficacy and tolerability of phage-treated burn wound infections, and it contributed to the establishment of a protocol for phage therapy (Jault et al., 2019). Only temporary accommodations were achieved. Although recommendations for further clinical trials were made, no substantial efforts have been made to advance the regulatory framework. Furthermore, if regulations are revised to accommodate phage therapy, questions arise regarding the position of phage product manufacturers on intellectual property rights. Specifically, is it feasible to patent and commercialize naturally occurring biological organisms, or is patentability limited to phage-antibiotic combinations and phage cocktails exhibiting unique antibacterial properties? To cater to the specific needs of individual patients, it may be necessary for phage cocktails to be trademarked and sold as distinct products, or perhaps such cocktails could be designed for subsequent modification to address particular patient requirements (Bhattarai et al., 2018). The lack of a clearly defined, commercially viable pathway for technology development may deter pharmaceutical companies from investing in the research and development of such therapeutics.

## 8 Combination therapy

Several *in-vitro* studies have shown that bacterial resistance is increasing day by day against phage therapy. There is a need to find an alternative therapeutic strategy to overcome bacterial resistance (Cao et al., 2015; Kumari et al., 2010; Tabassum et al., 2018). The combinational treatment, either the combination of antibacterial drugs with phages or a cocktail of phages, is a new approach to reducing bacterial resistance. Many researchers are using this new approach for the treatment of phage-resistant strains of *K. pneumoniae* (Chadha et al., 2016; Verma et al., 2009b; Gu et al., 2012). Chadha *et al*. observed the efficacy of a single phage in contrast to a phage cocktail in BALB/c mice for the treatment of *K. pneumoniae* B5055. The results of this showed that a phage cocktail (combination of Kpn1, Kpn2, Kpn3, Kpn4, and Kpn5) reduces the bacterial load in a shorter period as compared to individual phages. Another study confirmed these results by studying the efficacy of a single phage in contrast to a phage cocktail for the reduction of bacterial load. A phage cocktail (a combination of three lytic phages GH-K1, GH-K2, and GH-K3) that have different but overlapping host specificities for the treatment of *K. pneumoniae* strain K7. The results suggest that the co-culture of the phage cocktail and K7 decreased the production of phage-resistant variants of K7 and reduced the bacterial load as compared to a single phage. Furthermore, the phage cocktail induction in bacteremia mice infected by K7 increases the survival rate of mice and lowers the blood bacterial count as compared to individual phage induction in mice (Gu et al., 2012). Verma et al. explored the new strategy in combination therapy. They used a combination of ciprofloxacin and lytic phages for the treatment of *K. pneumoniae* biofilms. The authors found that this combination reduced the development of ciprofloxacin-resistant and phage-resistant strains of *K. pneumoniae* and has a greater effect against biofilms than individual treatments. These studies proved that combination therapy is more effective for the treatment of infectious diseases and for preventing the emergence of bacterial resistance (Verma et al., 2009b).

## 9 Human trials

The use of phages for clinical trials involving humans faced regulatory friction in the West as compared to Eastern Europe and the former Soviet Union, which used phages in healthcare units for many years (Kutter et al., 2010). For example, the Herzfeld Institute of Immunology and Experimental Therapy in Poland and the Eliava Institute of Bacteriophages, Microbiology, and Virology in Georgia produce and supply phage products for human use (Furfaro et al., 2018). The regulatory friction has slowed the use of phages for human trials. The unique nature of phages as self-replicating and evolving biological entities demands new rules and regulations for their production, safety, and use as compared to traditional therapeutic agents. The lack of these regulations overcomes their advancements as well as pharmaceutical companies also have a lack of interest due to the concept of personalized medicine related to phage therapy. The concept of personalized medicine makes the approval process more difficult and lengthier for human trials (Debarbieux et al., 2016). Despite these challenges, the Belgian government has stated the implementation of a magisterial phage regulatory framework. Pharmacists prepared non-authorized phage products based on physician prescriptions for individual patients. The phages can occasionally be used in Europe under Article 37, the Declaration of Helsinki (Pirnay et al., 2018). The use of phages for human trials has been carried out despite these hurdles. Dutch clinician successfully treated the renal transplant patient with a recurrent urinary tract infection (Gonzalez-Menendez et al., 2018) infected by extended-spectrum beta-lactamase (ESBL) *K. pneumoniae* with the combination of phages and meropenem. The treatment of meropenem alone failed to treat the infection. The patient got phage therapy from Eliava Institute, and the patient sustained infection-free after the therapy. This result proved that combination therapy was effective (Kuipers et al., 2019). Italian clinicians also successfully used the phage cocktail for the treatment of invasive infections MDR, KPC-3-harboring *K. pneumoniae* (ST307). Phages are used for the decolonization of bacteria from the gut. Gut decolonization is a critical step to prevent future infection and the growth of resistant bacteria. The results showed that this treatment has no adverse effects (Corbellino et al., 2020). A prospective study related to the use of phage cocktails and phage for the treatment of skin ulcers was performed in India. These ulcers were showing resistance against antibiotics due to bacterial biofilms. However, phages successfully treated the skin ulcer and eradicated the biofilm formation (Patel et al., 2021). These studies demonstrate the potential use of phage therapy for the treatment of resistant bacteria and to overcome the effect of antibiotic resistance. These studies showed that clinical success shows the effectiveness of phage therapy as an alternative approach to antibiotic treatments.

## 10 Utilization of phage-derived peptides

Utilization of phage-derived Peptides instead of whole phage provides another alternative therapy option for multidrug-resistant *K. pneumoniae*. This kind of therapy has the potential to be beneficial as it is more convenient and expeditious to get clinical authorization for the recombinant protein. These phage-derived proteins tend to be utilized in their intact form against MDR bacterial pathogens like *K. pneumoniae* or as an integral part of a combination strategy to replace or boost existing antibiotics. Phage-derived peptides are formed during the lytic cycle of phage and help in bacteriophage in several functions, such as phage adsorption to the bacterial cell wall, infectivity, replication, and release of phage progeny. There are various antimicrobial peptides against MDR *K. pneumoniae* that have potent antimicrobial activity (Majkowska-Skrobek et al., 2018). These phage-derived proteins are polysaccharide depolymerase, peptidoglycan hydrolases, and virion-associated lysins. Bacteriophages use endolysins, which are lytic enzymes, at the terminal stage of their multiplication cycle to hydrolyze the peptidoglycan in the bacterial cell wall, resulting in the rapid lysis and the release of their progeny (Matsuzaki et al., 2005).

Phage-derived peptides mostly include peptidoglycan hydrolase enzymes and polysaccharides depolymerases. These proteins act as enzymes and are commonly present in tail spikes and play an important role in effectively infecting bacteria after adsorption. Polysaccharide depolymerase catalyzes the bacterial cell wall's carbohydrates. On the other hand, peptidoglycan hydrolases attack the peptidoglycan layer and its breakdown, breaching the bacterial cell to get entry into the cell cytoplasm, where it deposits its genetic material (Drulis-Kawa et al., 2015). Three types of proteins, namely Spanin, holins, and endolysins, have been synthesized upon bacterial infection that play a critical role in making phage particles and bacterial cell wall lysis to release virions. Holins are hydrophobic and involved in the permeability of the inner cell membrane and make way for the other protein endolysin to attack the cell wall and destroy the peptidoglycan layer. Spanins are commonly involved in the disruption of Gram-negative bacterial cell membranes. Following this lysis, bacterial cell death occurs due to osmolysis.

### 10.1 Polysaccharide depolymerase

The capsule is the most significant cause of immune evasion in *K. pneumoniae*. Therefore, it has been the primary target for recombinant phage protein studies. Tail fibers of phage KP32 have an additional Tail tubular protein A (TTPA) that exhibits the activity of polysaccharide depolymerase (Pyra et al., 2017). An analogous method of cloning, expression, and spot test was performed (Pan et al., 2017). In this study, nine polysaccharide depolymerases have been identified. These were produced by phage ΦK64-1 isolated against *K. pneumoniae*. Each of these enzymes displayed specific activity in different capsular types with a wide range of hosts. This finding is intriguing since it not only validates the role of enzymes in determining the host specificity of phages. It also supports the concept of recombinant enzymes, which can target various strains of *K. pneumoniae*. In addition, many in vivo studies have been conducted to examine the impact of polysaccharide depolymerase upon infection of *K. pneumoniae*. A polysaccharide depolymerase derived from KP36, named depoKP36, was identified, cloned, and expressed in *E. coli* (Majkowska-Skrobek et al., 2016). This enzyme developed haloes in spot test on the bacterial lawns culture on an agar plate. Furthermore, the efficacy of this enzyme was evaluated in treating infection in *G. mellonella*. All larvae died with no treatment, while up to 40% lived upon treatment with enzymes after infection. Even when the enzyme was introduced to the bacteria before infection, the mortality rate was only 23%. These findings demonstrate that the decapsulating effect of depoKP36 on *K. pneumoniae* resulted in a lower ability of *K. pneumoniae* to deal with the immunological response.

### 10.2 Endolysin

Considerable research has been carried out on endolysins for their potency against Gram-positive bacterial cell walls. This is because Gram-negative bacterial cells, like *K. pneumoniae* possess an outer membrane, which prevents endolysin activity when spanins are not present. However, some recently conducted studies have also shown positive results on the potency of these enzymes against Gram-negative bacteria. Recombinant endolysin of KP27 (*K. pneumoniae* phage derived) effectively infects the other gram-negative bacteria as well. The findings indicate that in order to effectively tackle *K. pneumoniae*, therapies based on endolysin have to be complemented by an outer membrane permeabilizing compound (Majkowska-Skrobek et al., 2016).

Artilysin or Synthetic lysins were created by combining a phage endolysin and an outer-membrane-destabilizing peptide to address the demand for extra outer membrane permeabilizing agents for the management of Gram-negative bacteria infections (Briers et al., 2014b). Although artilysins targeted precisely for *K. pneumoniae* are yet to be identified, they have been effectively produced for the treatment of *P. aeruginosa* infections (Briers et al., 2014a) and *Acinetobacter baumannii* infections (Defraine et al., 2016). This method enables the invention of synthetic endolysins for human use against both multidrug-resistant gram-negative species and *K. pneumoniae*.

## 11 Phage safety profile

In comparison to other antibacterial agents that include antibiotics, phages can exhibit a wider range of modes of action and, in many situations, are safer. Because of their prevalence and our frequent exposure to them in the environment (Sulakvelidze et al., 2001), phages are generally regarded as safe, and they have been employed widely in some parts of the world with no reports of negative effects (Pirnay et al., 2011). Despite this hopeful outlook, the safety of phage therapy must be confirmed by sophisticated scientific investigations. One of the primary hurdles for phage therapy is the novel safety implications of using self-replicating biological organisms in humans. For instance, toxin encoding and antibiotic-resistant genes carried by the phages can be transferred to the other bacteria through the transduction process (Colavecchio et al., 2017; Abedon, 2018). The proper screening of the potentially dangerous genes is done throughout the process when investigating the phages for therapeutic purposes. Phages containing hazardous products can be purified during the formulation process. Bacteriophages, especially phage cocktails, have little effect on the commensal microbiota because of their high specificity toward the bacteria, and they only infect a small number of specific bacterial species. The possibility of phages encoding harmful genes can be prevented by avoiding the usage of temperate phages. Another obstacle for phage therapy is figuring out how to capitalize on these beneficial characteristics in light of current regulatory standards, as well as how phages can fit into the current economic frameworks that support the distribution and usage of antibacterial drugs (Townsend et al., 2021). To present, just a few clinical trials have been conducted, in all these studies, no major adverse effects were seen.

An inherent challenge in phage treatment is the safety concerns related to the application of self-replicating biological organisms in human beings. As an example, it is clear that phages can transport AMR genes (Colavecchio et al., 2017) and toxins encoding genes (Strauch et al., 2001) from one bacterium to another through a process known as transduction. Achieving accurate characterization is crucial when evaluating phages for uses in therapy, and the detection of potentially deleterious genes can frequently be tracked throughout this procedure. Nevertheless, the lack of detrimental genes do not ensure the safety and reliability of phage.

The inherent characteristic of a lytic bacteriophage is to proliferate itself by infecting bacterial cells. Although the main objective of phage treatment is to achieve this, little investigation has been carried out on the possible negative impact of this behavior. A key aspect to consider is that phages targeting a wide variety of hosts, or those seen in a phage cocktail, frequently qualify as more suitable for phage treatment. The latest study demonstrates that the introduction of a monophage therapy in mouse microbiota could indeed disrupt the composition of the microbiome (Hsu et al., 2019). How much of an effect would the treatment usage of phages likely have on the indigenous microbiota of human beings? Does using mono phages therapy, with a limited host range, provide a safer approach for mitigating disturbance of the microbiome? If such is the case, phage therapy will depend on the very precise identification of the targeted bacteria and the availability of the appropriate phage for treatment. On the other hand, this specific adverse reaction may be considered tolerable, comparable to the situation with present antibiotic therapies. Furthermore, there is a lack of clinical studies that have evaluated the safety of phage treatment in human beings. In addition, the existing studies have been characterized by restricted numbers of participants and have often depended on data supplied by patients (Furfaro et al., 2018).

## 12 Future perspective

Emerging advancements in recombinant enzyme technology have shown promising alternative therapeutic options against resistant strains of *K. pneumoniae*. The capsular degradation is the most critical by polysaccharide depolymerase, making these enzymes a potent therapeutic agent against *K. pneumoniae* and its associated biofilms. Synthetic endolysins can function as autonomous antimicrobial agents against infections. Continued investigation is necessary in this field to fully understand the capabilities of phage-derived recombinant proteins in harnessing bacterial infections. One more perspective to be covered in the future is to investigate the methods by which these proteins reduce bacterial growth and eradicate infection. This will significantly enhance the potential of this endolysin. One significant benefit of engineered proteins for human use is the presence of clearly defined and established norms and regulations in Europe and the UK that cover their manufacturing, safety, and implementation rules. On the other hand, whole phage therapy lacks such standard protocols.

## 13 Conclusion

The growing rate of infections from hospitals and communities produced by multidrug-resistant *K. pneumoniae* is swiftly emerging as a public health threat globally. Along with MDR strains of *K. pneumoniae*, the emergence of hypervirulent strains is also a worldwide issue. *K. pneumoniae* has become an important concern over its non-resistant analogs of today are more than a century old. In reaction, research endeavors have started to investigate a previously neglected method of treating bacterial infections, namely bacteriophage treatment. It is becoming very evident that bacteriophages and their proteins have potential therapeutic effects against these MDR strains and can be used as an alternative approach over conventional antibiotics toward which pathogens become resistant. Nevertheless, the arena of phage treatment is still in its early stages and is limited to challenges. But there is still hope for some innovative approach can be recognized to overcome these obstacles.
